# Resequencing the susceptibility gene, *ITGAM*, identifies two functionally deleterious rare variants in systemic lupus erythematosus cases

**DOI:** 10.1186/ar4566

**Published:** 2014-05-21

**Authors:** Amy L Roberts, Ellen RA Thomas, Shriram Bhosle, Laurence Game, Olga Obraztsova, Timothy J Aitman, Timothy J Vyse, Benjamin Rhodes

**Affiliations:** 1 Department of Medical and Molecular Genetics, and Inflammation, Infection and Immunity, King’s College London, London SE1 9RT, UK; 2 MRC Clinical Sciences Centre, Imperial College London, London, UK

## Abstract

**Introduction:**

The majority of the genetic variance of systemic lupus erythematosus (SLE) remains unexplained by the common disease-common variant hypothesis. Rare variants, which are not detectable by genome-wide association studies because of their low frequencies, are predicted to explain part of this ”missing heritability.” However, recent studies identifying rare variants within known disease-susceptibility loci have failed to show genetic associations because of their extremely low frequencies, leading to the questioning of the contribution of rare variants to disease susceptibility. A common (minor allele frequency = 17.4% in cases) nonsynonymous coding variant rs1143679 (R77H) in *ITGAM* (CD11b), which forms half of the heterodimeric integrin receptor, complement receptor 3 (CR3), is robustly associated with SLE and has been shown to impair CR3-mediated phagocytosis.

**Methods:**

We resequenced *ITGAM* in 73 SLE cases and identified two previously unidentified, case-specific nonsynonymous variants, F941V and G1145S. Both variants were genotyped in 2,107 and 949 additional SLE cases, respectively, to estimate their frequencies in a disease population. An *in vitro* model was used to assess the impact of F941V and G1145S, together with two nonsynonymous *ITGAM* polymorphisms, A858V (rs1143683) and M441T (rs11861251), on CR3-mediated phagocytosis. A paired two-tailed *t* test was used to compare the phagocytic capabilities of each variant with that of wild-type CR3.

**Results:**

Both rare variants, F941V and G1145S, significantly impair CR3-mediated phagocytosis in an *in vitro* model (61% reduction, *P* = 0.006; 26% reduction, *P* = 0.0232). However, neither of the common variants, M441T and A858V, had an effect on phagocytosis. Neither rare variant was observed again in the genotyping of additional SLE cases, suggesting that there frequencies are extremely low.

**Conclusions:**

Our results add further evidence to the functional importance of *ITGAM* in SLE pathogenesis through impaired phagocytosis. Additionally, this study provides a new example of the identification of rare variants in common-allele-associated loci, which, because of their extremely low frequencies, are not statistically associated. However, the demonstration of their functional effects adds support to their contribution to disease risk, and questions the current notion of dismissing the contribution of very rare variants on purely statistical analyses.

## Introduction

Systemic lupus erythematosus (SLE) is a phenotypically heterogeneous autoimmune disease resulting from the breakdown of immune tolerance. SLE has a strong and complex genetic component, as shown by estimates of twin concordance [1]. Although 52 genetic loci now are independently associated with SLE, the vast majority of which are products of the genome-wide technologic advancements of recent years, only an estimated 16% of the total variance is thought to be explained [2,3] (J. Bentham, D. L. Morris, *et al*. 2014, unpublished data].

The genome-wide association study (GWAS) era has uncovered a plethora of common alleles, mostly of modest effect sizes (odds ratio (OR), <2), associated with complex diseases under the common disease-common variant (CDCV) hypothesis [4]. Yet for most of these diseases, as with SLE, the amount of remaining unexplained genetic variance (the so-called missing heritability) is higher than initially anticipated [5-7]. Rare variants with larger effect sizes (OR, ≥2), but present at frequencies below the detectable threshold of GWAS (<5%), have been hypothesized to explain part of this ”missing” genetic effect [8,9]. Although they are individually rare, they are collectively common and therefore may make a significant contribution to disease risk on a population level.

Because of their low frequency and incomplete penetrance, individual rare variants may never reach statistically significant levels of association, irrespective of very large sample sizes [10]. Furthermore, the relatively high *de novo* mutation rate in humans [11], means that chromosomes of unaffected individuals will also harbor rare, or even private, mutations that may or may not influence disease risk. Genome- or exome-wide sequencing may uncover many new variants, but determining which of these are functionally relevant is not easy by genetic analysis alone [12]. One approach is to use the notable common variant discoveries of GWAS to focus on resequencing candidate genes that are already known to contain common disease-risk variants. Many studies have now identified additional rare variants in GWAS-associated loci [13,14]. The largest of such studies focused on the coding exons of 25 autoimmune GWAS-associated loci and concluded that a negligible affect from rare variants was present [15]. Ultimately, however, functional analyses of rare variants will best enable us to understand the importance of associated loci in disease pathogenesis and add confirmatory evidence to disease biology paradigms established by the functional effect of common variants.

SLE is robustly associated with *ITGAM* (ENSG00000169896), which encodes CD11b [16]. Through non-covalent binding with CD18, this forms the heterodimeric integrin complement receptor 3 (CR3; Mac-1; CD11b/CD18), expressed on phagocytes and NK cells [17]. Despite the high degree of linkage disequilibrium (LD) across the *ITGAM* locus in European populations, transancestral mapping provided evidence that the common nonsynonymous polymorphism rs1143679, encoding an arginine-to-histidine amino acid change at codon 77 of CD11b (R77H), was the true causal SNP [18]. Subsequently, functional evidence supported this genetic association, showing that R77H impairs many functions of the CR3 receptor, including the phagocytosis of iC3b-coated targets by *ex vivo* monocyte-derived macrophages [19], neutrophils [20], and *in vitro* models [21]. In addition to rs1143679, speculation has occurred about a secondary independent SLE association within *ITGAM* due to a second nonsynonymous variant rs1143683 (A858V) [22]. Because precedence exists for the presence of multiple effects in risk loci of complex diseases, this requires further evaluation [23].

Here we report the identification of two novel, case-specific rare variants in *ITGAM* after resequencing of this locus in cases of European ancestry. Both variants, F941V and G1145S, were functionally damaging, as measured by an *in vitro* model of CR3-mediated phagocytosis, but the common A858V (rs1143683) variant was functionally neutral. Furthermore, neither F941V nor G1145S was observed in the follow-up genotyping of 2107 and 949 SLE cases, respectively. This study shows the importance of multiple approaches to the analysis of rare variants, and the need for better methods to estimate contributions, before we dismiss the importance of this large body of genetic variation.

## Methods

### The 454 sequencing and analysis

Genomic DNA from 73 SLE patients meeting American College of Rheumatology criteria underwent whole-genome amplification (Qiagen Repli-G, Hilden, Germany). Ethical approval was given by London Multicentre Research Ethics Committee, and participants gave written consent. Standard (KOD polymerase, Merck) and long-range (Sequalprep, Invitrogen, CA, USA) PCR reactions were carried out in 13 amplicons, giving products between 600 base pairs and 4 kb in length, covering 24 kb of the *ITGAM* gene, including all 30 exons (see Additional file 1). The PCR products were run on an agarose gel and purified by using the Qiagen MinElute 96 UF PCR Purification Kit. Seven amplicons greater than 1.5 kb were pooled and sheared to fragments of 500 to 800 bp on the Covaris E210. The six short PCR products were added to the sheared products, and each pool was tagged with a unique DNA barcode by using the Parallel Tagged Sequencing (PTS) protocol [24], starting at the blunt-end repair step. The barcoded PCR products were quantified by using the Invitrogen Quant-iT Picogreen dsDNA kit and combined into a single pool by using 10 ng of each tagged sample.

Dephosphorylation and restriction digestion was carried out according to the PTS protocol, and the pool of sheared and indexed PCR products was taken through 454 Titanium library preparation according to the manufacturer’s protocol (GS FLX Titanium General Library Preparation Method Manual, v5.3.2), starting at step 3.5. Library quality was checked by using an RNA Pico 6000 assay on the Agilent Bioanalyser and quantified by using a RiboGreen assay (Thermo Fisher Scientific, MA, USA) with the Nanodrop 3300 fluorospectrometer. The library was diluted to 1 × 10^8^ molecules/μl, amplified and sequenced according to the manufacturer’s protocol for 454 GS FLX sequencing. With 896,206 reads that passed filters, an average coverage of 105× was achieved; 88% of the samples were covered at a level sufficient for variant calling (more than 10 reads) in at least 80% of the target region.

Additional PCRs (KOD polymerase, Merck, Darmstadt, Germany) were generated covering exons 8, 13 and 14 due to low coverage in the 454 data. Capillary sequencing of these amplicons used the 3730xl platform (Applied Biosystems, CA, USA).

The 454 sequence read demultiplexing used ”untag” software [24]; base calling used Pyrobayes [25]; and alignment to the reference genome used BWA [26]. Variant calling was carried out by using GATK [27] without duplicate read removal, by using hard filters: coverage at least 10-fold; genotype quality threshold, 50; SNP quality threshold, 30; and allele balance threshold, 0.75.

The presence of novel variants was confirmed independently by PCR amplification of native genomic DNA followed by capillary sequencing (see Additional file 2). G1145S was genotyped in 949 additional SLE cases with capillary sequencing by using the following primers: 5′ GGCTTCTTCAAGCGGCAATA and 5′ GTCCTGTCGGGGATACTTCG. F941V was genotyped in 2,107 additional SLE cases on an Illumina Custom 384 chip.

### Site-directed mutagenesis

10 ng of a pcDNA3.1-CD11b vector (Life technologies, Paisley, UK) was mutated to contain one of four *ITGAM* variants by using the PCR-based QuikChange II XL Site-Directed Mutagenesis kit (Agilent Technologies, Stockport, UK), followed by transformation of XL-10 Gold Ultra-competent cells, as per the manufacturer’s instructions. The primers used for the introduction of each of the variants were as follows: M441T 5′-gcagaacactggcacgtgggagtccaacg-3′ and 5′-cgttggactcccacgtgccagtgttctgc-3′; A858V 5′-ccgaagtgtctggggtcttgaagagcaccag-3′ and 5′-ctggtgctcttcaagaccccagacacttcgg-3′; F941V 5′-tctccactaaatatctcaacgtcacggcctcagagaat-3′ and 5′-attctctgaggccgtgacgttgagatatttagtggaga-3′; G1145S 5′-atgagtgaagggagtcccccggggg-3′ and 5′-cccccgggggactcccttcactcat-3′.

### Transient transfections

COS-7 cells (ATCC, USA) were maintained in 10% FBS DMEM (Life Technologies); 1 × 10^6^ cells were co-transfected with one of five pcDNA3.1-CD11b vectors (WT, M441T, A858V, F841V, or G1145S) together with 1.25 g pcDNA3.1-CD18 by using nucleofection (Nucleofector II; Lonza, Basel, Switzerland). In duplicate, 40 × 10^4^ cells were added to an acid-cleaned coverslip in a 24-well plate. The remaining transfected cells were cultured for expression screening with flow cytometry.

### Flow cytometry

One or two days after transfection, cell-surface expression of CR3 was measured by using a PE-conjugated anti-CD11b (clone ICRF44) antibody and PE-conjugated isotype control. Transfected cells were stained in the dark for 30 minutes on ice and processed on a BD FACS Canto II flow cytometer. The percentage of CR3-positive cells was estimated, and the mean fluorescence intensity (MFI) measured, by using FlowJo software v.7.6.4. A paired *t* test was used to compare the MFI of the CR3-positive cell population. No significant difference in cell-surface expression of CD11b was observed between WT (*n* = 5, mean ± SD; 14,445 ± 6,982) and M441T (15,017 ± 7,993), A858V (12,377 ± 5,130), F941V (14,327 ± 4,358), or G1145S (12,767 ± 5,028) CD11b transfected cells.

### Phagocytic assay

This was carried out on the same day as the flow-cytometry expression analysis. The method used is identical to that in our previously published work [19] and has been used extensively as an *in vitro* model for phagocytic receptors [28,29]. In summary, sheep red blood cells (sRBCs) were coated with anti-sheep IgG at room temperature for 1 hour, followed by incubation at 37°C for 20 minutes with C5-depleted serum to form iC3b-opsinized sRBC (sRBC_iC3b_). Transfected COS-7 cells were challenged with sRBC_iC3b_ for 30 minutes at 37°C. External sRBC_iC3b_ were labeled with an Alexa-488 conjugated anti-IgG antibody and, after fixation and permeabiliation, both internal and external sRBC_iC3b_ were labeled with an Alexa555-conjugated anti-IgG antibody. COS-7 nuclei were stained with DAPI. A Zeiss Axiophot fluorescent microscope was used visually to analyze the coverslips.

### Analysis

Associated RBCs are double-stained with Alexa-488 and Alexa-555 and are therefore visible under both fluorescent filters. A phagocytic event was characterized by the absence of the Alexa-488 stain. We measured the association index (AI, mean number of engaged sRBC_iC3b_/100 COS cells), phagocytic index (PI, mean number of phagocytosed sRBC_iC3b_/100 COS cells), and percentage phagocytosis (%P, mean percentage phagocytosis/COS cell). A mean number of 67 COS-7 cells were counted from at least two coverslips per assay. Comparisons were made between WT and variants by using paired two-tailed *t* tests on Prism5 software (GraphPad, CA, USA).

## Results

### Two novel non-synonymous variants discovered through *ITGAM* resequencing

We used a candidate-gene approach and resequenced *ITGAM* by using the high-throughput 454 GS FLX platform in 73 SLE cases in search of putative rare variants. Then 24 Kb of the *ITGAM* locus was sequenced, covering all 30 exons and every intron/exon boundary. Two rare novel variants were identified in one patient sample each, F941V and G1145S (Table 1), and were confirmed by capillary sequencing after PCR amplification of native genomic DNA (Additional file 2). F941V was further genotyped on an Illumina Custom 384 Chip in 2,107 SLE cases of European ancestry. G1145S did not pass QC analysis of this genotyping method, possibly because of its close proximity (3 bp) to the common polymorphism P1146S, and so was genotyped with capillary sequencing in 949 SLE cases of European ancestry. Neither variant was observed again in these additional cases; therefore, we can estimate the minor allele frequencies of these variants to be F941V, ≤0.0002, and G1145S, ≤0.0005.

**Table 1:** **Summary of common polymorphisms and rare variants used for *in vitro *** **study**

**Variant**	**Chromosomes from resequencing study with minor allele (total = 146)**	**PolyPhen-2**	**SIFT**
M441T	21	Benign (0)	Tolerated (0.63)
A858V	33	Benign (0.001)	Tolerated (0.35)
F941V	1	Probably damaging (1.00)	Damaging (0)
G1145S	1	Benign (0.319)	Tolerated (0.5)

PolyPhen-2 [30] categorizes mutations as benign, possibly damaging, or probably damaging, based on pairs of false-positive rate (FPR) thresholds. SIFT [31] predicts variants to be deleterious, given an output value <0.05.

**Figure 1:**
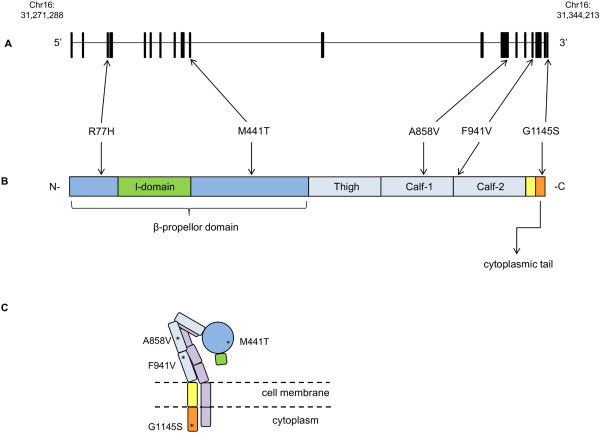
***ITGAM *transcript, CD11b primary protein domains, and CR3 protein. (A)***ITGAM* genomic organization showing relative positions of common polymorphisms (R77H, M441T, A858V) and novel rare variants (F941V, G1145S). **(B)** Primary protein domains of CD11b and the relative location of the missense variants. The β-propellor domain is shown in dark blue; the ligand-binding I-domain is shown in green; light blue represents the other extracellular domains; the transmembrane domain is shown in yellow, and the short cytoplasmic tail is represented in orange. **(C)** A cartoon diagram of the CR3 protein (Protein domain ratios = extracellular/transmembrane/cytoplasmic = 1:6:6). The same colors as **(B)** are used to represent the various domains; CD18 is shown in light purple. Asterisks are used to indicate the position of missense variants used in the functional studies: M441T, A858V, F941V, and G1145S.

CD11b codons 941 and 1145 are located in the membrane-proximal extracellular domain and the short cytoplasmic tail of the protein, respectively (Figure 1). Table 1 shows the *in silico* estimates of pathogenicity by using PolyPhen and SIFT. F941V was predicted to be deleterious, and G1145S was predicted to be benign, by both algorithms.

We used two large-scale publicly available sequencing project datasets to screen *ITGAM* on the chromosomes of healthy controls of European ancestry. Neither variant, F941V or G1145S, is described in the 1,000 chromosomes of European ancestry in the 1,000 Genomes project [32], nor are they described in the 8,600 European-American chromosomes of The National Heart Lung and Blood Institute (NHLBI) Exome Variant Server [33]. Therefore, both variants are novel, and, if not private, they are present at a frequency below 0.0001 in healthy individuals of European ancestry. It is worth noting that neither variant is found in the other populations included in the databases. This was expected, given that rare variants will be population specific.

**Figure 2:**
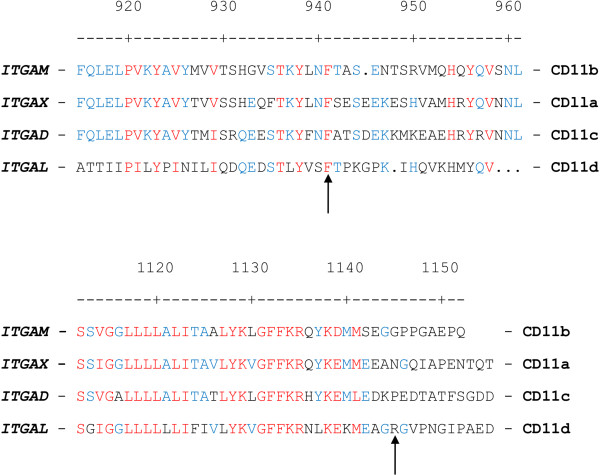
**Comparisons of CD11 amino acid sequences.** CD18 pairs with CD11b, CD11a, CD11c, and CD11d, and together, these make up the β_2_ integrin family. The four CD11 protein sequences (encoded by *ITGAM, ITGAX, ITGAD,* and *ITGAL*) were aligned by using MutAlin. The numbers indicate the CD11b codons, and all other sequences are aligned relative to this. F941 and G1145 of CD11b are indicated by arrows. The phenylalanine at codon 941 is present across all four CD11 polypeptides; the glycine at codon 1145 is not found in any other CD11 polypeptide.

MultAlin [34,35] was used to compare the protein sequences of the four CD11 molecules that pair with CD18 as part of the β_2_ integrin family (Figure 2). The four proteins, CD11a, CD11b, CD11c, and CD11d, are encoded by *ITGAX, ITGAM, ITGAL,* and *ITGAD,* respectively. The phenylalanine (F) residue at CD11b codon 941, which is located in the membrane-proximal extracellular domain, is conserved across all four polypeptide sequences. The Glycine (G) residue at CD11b codon 1,145 is not shared with any of the other three CD11 proteins. No homology occurs at this relative position among any of the CD11 amino acid sequences. The short cytoplasmic tails of the CD11 molecules vary in length and sequence, and the differences observed are likely to be crucial for their varying functions, and/or intracellular signaling properties. Data from the UCSC Genome Browser [36] indicated that both CD11b residues, F941 and G1145, are highly conserved across mammalian species (data not shown).

### *In vitro* analyses of rare and common variants

We used transiently transfected COS-7 cells to measure the effect of newly identified *ITGAM* rare variants on the binding and phagocytosis of iC3b-coated sheep red blood cells (sRBC_iC3b_), in addition to CR3 cell-surface expression. Additionally, we included the two common polymorphisms M441T (rs11861251) and A858V (rs1143683), which are in low (*r*^*2*^ = 0.017) and relatively high (*r*^*2*^ = 0.551) LD, respectively, with R77H in European populations, in our *in vitro* analyses. LD scores were calculated with 1000 Genomes Pilot 1 CEU data set by using SNAP Pairwise LD [37].

**Figure 3:**
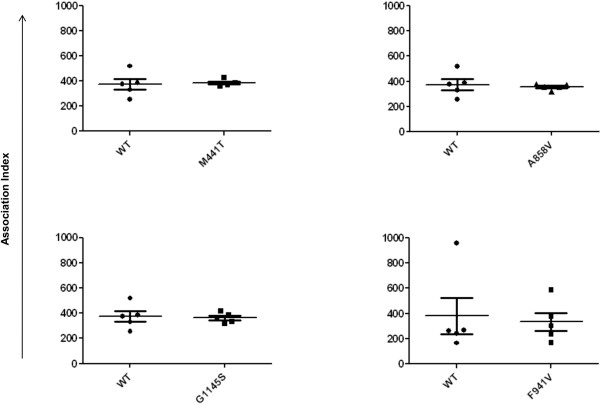
**Association of CR3-expressing COS-7 cells with sRBC**_**iC3b**_**.** Association Index = mean number of associated (internal and external) sRBC_iC3b_/100 COS-7 cells. No significant difference was observed between WT and M441T (*P* = 0.79), A858V (*P* = 0.65), G1145S (*P* = 0.85), or F941V (*P* = 0.61).

To test whether the nonsynonymous variants affected iC3b binding, we measured the ability of transiently transfected COS-7 cells to associate with iC3b-opsinized sheep red blood cells (sRBC_iC3b_) by using an Association Index. No significant difference was observed when variant CD11b was compared with WT, indicating that none of the four variants (M441T *P* = 0.79; A858V *P* = 0.69; F941V *P* = 0.61; G1145S *P* = 0.85) affects the iC3b-binding ability of CR3 (Figure 3). Given that the four variants are located outside the iC3b-binding site of the protein (Figure 1), this was an expected result.

**Figure 4:**
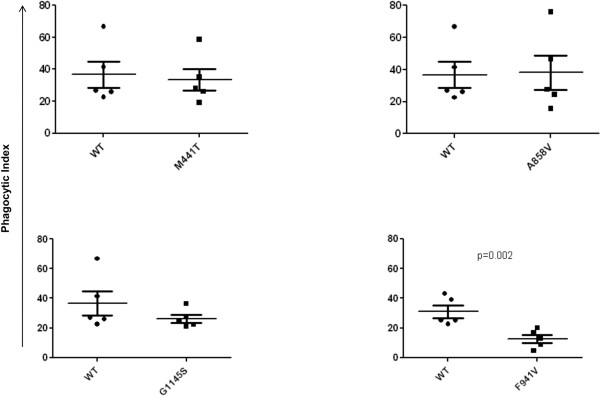
**Phagocytosis of sRBC**_**iC3b **_**by CR3-expressing COS-7cells as measured with the Phagocytic Index.** Phagocytic Index, mean number of internal sRBC_iC3b_/100 COS-7 cells. F941V significantly impairs phagocytosis (*P* = 0.002). No significant difference observed between WT and either common polymorphism M441T (*P* = 0.74) and A858V (*P* = 0.92), or the rare variant G1145S (*P* = 0.37).

**Figure 5:**
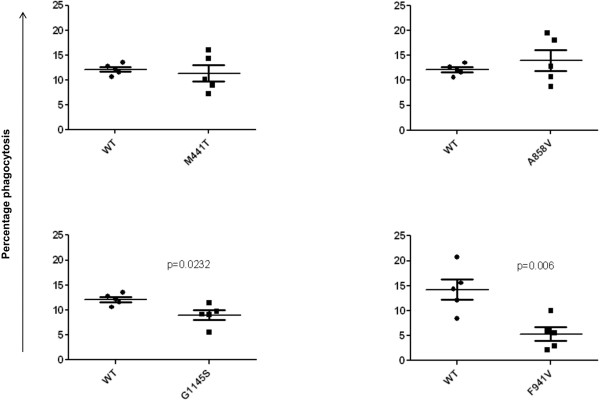
**Phagocytosis of sRBC**_**iC3b **_**by CR3-expressing COS-7cells as measured with percentage phagocytosis.** Percentage phagocytosis, mean percentage phagocytosis/COS-7 cell. Both rare variants F941V and G1145S significantly impair phagocytosis (*P* = 0.006 and 0.0232, respectively). No significant difference was observed between WT and either common polymorphism M441T (*P* = 0.70) and A858V (*P* = 0.47).

F941V significantly impaired the Phagocytic Index (*P* = 0.002; Figure 4) and the percentage phagocytosis (*P* = 0.006; Figure 5), and the G1145S variant significantly impaired percentage phagocytosis (*P* = 0.0232; Figure 5), when compared with the WT (see Additional file 3). Neither of the two common polymorphisms had an effect on the phagocytosis of sRBC_iC3b_ as measured by the Phagocytic Index (M441T, *P* = 0.74; A858V, *P* = 0.92; Figure 4) and percentage phagocytosis (M441T, *P* = 0.70; A858V, *P* = 0.47; Figure 5).

**Figure 6:**
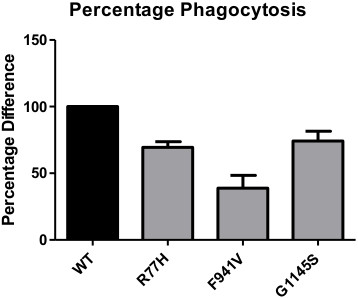
**Percentage reduction in phagocytosis of CD11b variants compared with WT.** Comparison of the functional impact of the two rare variants with that of the SLE-associated R77H (previously published data [19]). Mean and SEM are shown.

The same transient transfection COS-7 model had previously been used by our group to demonstrate the deleterious effects of the SLE-associated missense polymorphism, R77H, on iC3b-dependent phagocytosis [19]. We used the mean reduction in percentage phagocytosis to compare the results of this study with that of the previous R77H study (Figure 6). As seen here, G1145S (26%) has a comparable magnitude of effect to that of R77H (31%), whereas F941V (61%) has an even greater effect on phagocytosis.

## Discussion

Through resequencing *ITGAM* in SLE patients of European ancestry, we identified two novel, case-specific variants. The *in vitro* model shows that both these variants, G1145S and, more strongly, F941V, impair CR3-mediated phagocytosis of iC3b-coated targets. The observed detrimental impact on protein function is of a similar (G1145S), or greater (F941V), magnitude to that of the common SLE-associated polymorphism R77H (which was associated with a 31% reduction in phagocytosis in an earlier article) [19]. Two factors support the importance of our data: first, they add further evidence for the coexistence of common and rare variants in genetic loci predisposing to the same disease biology, and second, they emphasize the role of underfunctioning *ITGAM* variants in SLE pathogenesis.

The targeted resequencing of loci harboring disease-associated common polymorphisms has successfully identified novel rare variants in a number of other complex genetic diseases such as inflammatory bowel disease, psoriasis, and asthma [13,14,38]. Recent studies in schizophrenia [39] and epilepsy [10] identified rare variants through whole-exome sequencing but failed to identify a single variant that reached study-wide significance, despite large-scale genotyping in follow-up cohorts. The schizophrenia study reported that 23% of case-specific variants from the initial sequencing cohort were not observed again in the follow-up cohort [39]. As demonstrated by these two studies, individual rare variants may never reach statistical significance for disease association. In our follow-up genotyping, 2,107 and 949 additional SLE cases were found to be wild-type for F941V and G1145S, respectively.

To assess the contribution of these novel *ITGAM* variants to disease biology, we additionally estimated their functional impact. We were able to do this by using a robust functional assay [28] that had previously proven successful in delineating function of the common *ITGAM* R77H variant [19]. Our work does not contribute to the estimate of overall genetic variance explained for SLE, but it does add evidence to the functional importance of the *ITGAM* locus to SLE susceptibility.

Furthermore, this study is another example of rare variants located in genetically associated loci whose contribution to disease risk cannot be estimated by statistical models alone because of their extremely low frequencies. However, given the demonstrated functional impact of these variants, their contribution cannot be dismissed. A case report of a CR3 defect in an SLE patient was previously assumed to be a result of a rare genetic variant [40]. Until additional methods of analysis are available, the contribution of rare variants to complex diseases should not be dismissed on the basis of non-significant statistical associations alone.

Although we observed clear functional similarities between the novel rare variants and R77H, the magnitude of this functional effect was different between variants, being stronger for variant F941V than for G1145S or common variant R77H. This raises an important point about the rare variant paradigm, which is often represented as a direct negative correlation between allele frequencies and disease risk [41]. Our *in vitro* model indicates that within a single gene, functional variants may vary considerably both in frequency (MAF = 0.2 versus MAF ≤0.0005) and functional magnitude.

Defective phagocytosis is a robust paradigm in SLE biology [42], but of course may not be the only mechanism by which impaired CR3 function predisposes to SLE risk. It is possible, then, that F941V and/or G1145S have variable effect sizes on other CR3 functions, such as the inhibition of Toll 7/8-induced proinflammatory cytokines; however, in the absence of *ex vivo* cells from multiple healthy individuals carrying these mutations, or additional suitable *in vitro* assays, we lack a sufficient model for this [19,43]. We recognize that it is difficult to estimate directly the contribution of these rare variants to SLE risk; however, we believe that because their impact on phagocytosis is equal to, or greater than, that of R77H, their effect should not be dismissed. This risk is likely to be modest (as it is for R77H), supported by the identification of F941V, which has the greatest impact on CR3 function, in unaffected family members through capillary sequencing.

Finally, both patients with *ITGAM* rare variants described here were included as part of a recent SLE GWAS (J. Bentham, D. L. Morris, *et al*., 2014, unpublished data) and their risk scores (allele count*ln(OR)) fall within 1 SD of the mean risk score for all cases (personal communication). If a relatively small number of rare variants were to contribute to large amount of the genetic variance of SLE, we might expect the two patients’ risk scores, obtained from their genomic composition of associated common variants, to be relatively low. Together with the comparable functional impact of G1145S and R77H, our work suggests that some individual rare variants may contribute less to the genetic variance of complex diseases than is currently hypothesized, but it is possible that, on a population-wide scale, the presence of individual rare variants with weak effect size is a common phenomenon and, in total, accounts for much missing heritability, despite the current lack of appropriate statistical tools. As we have demonstrated, if they were located in known susceptibility loci and if the right functional assay were available, they might be relatively easy to find. The majority, however, are likely to remain undiscovered.

As well as demonstrating a general aspect of rare-variant discovery, our data also expand on our specific understanding of the role of *ITGAM* variants in SLE pathogenesis. First, we have resolved some uncertainty in the genetic literature concerning the possible additional functional effect of *ITGAM* SNP rs1143683 (A858V). SNP rs1143683 (A858V) and rs1143679 (R77H) are in relatively high LD (*r*^*2*^ = 0.55) in European populations, and rs1143683 has been suggested as a secondary independent association signal within *ITGAM* in a Thai population [22]. We wished to clarify this issue by including the A858V polymorphism in our *in vitro* model, along with another polymorphism, rs11861251 (M441T), which is not a part of the same disease-associated haplotype (*r*^*2*^ = 0.017), which we envisaged as a negative control. We found that neither the A858V nor the M441T mutations affect the CR3-mediated phagocytosis of iC3b-coated targets.

A recent publication demonstrated a functional impact of *ITGAM* polymorphisms on *ex vivo* neutrophil functions, independent of R77H [44]. There is perfect LD between rs1143683 (A858V) and rs1143678 (P1146S), and for this reason, the functional impact of these variants could be analyzed only on a haplotype basis. Even though they report a significant reduction in phagocytosis in individuals with A858V/P1146S variant neutrophils, they hypothesize that the affect is attributable to the less-conservative P1146S amino acid change. Our results support this hypothesis of the functional neutrality of A858V. Of particular interest, the patient who harbors the G1145S rare variant is also heterozygous for the P1146S polymorphism. Through using the IGV program to view aligned 454 sequencing reads, we can see that the G1145S rare variant and P1146S polymorphism are in phase on the chromosome. G1145S had the least severe impact on phagocytic function (26% reduction); however, a functional alteration to two sequential amino acids could indeed have an impact greater than the additive effect of the G1145S and P1146S variants considered independently. This patient is also heterozygous for the associated R77H polymorphism (of note, the patient with F941V is homozygous WT), so it is possible that the CR3 functions of this person are severely affected *in vivo*. The phenomenon of an additive functional effect of both rare and common variants within the same polypeptide molecule is of particular interest when considering the contribution of rare variants to complex disease susceptibility.

Our functional results also emphasize that impaired phagocytosis of iC3b opsonized particles, a consequence of defective ”outside-in” signaling is one, but clearly not necessarily the only, functional mechanism underlying the association between specific *ITGAM* risk variants and SLE. It is notable that all three functional variants, R77H, F941V, and G1145S, are located in different domains of the CD11b polypeptide (Figure 1) but have similar effects on signaling. None appears directly to influence known ligand-binding sites [45], but clearly specific amino acids throughout CD11b, from the N-terminal β-propeller (R77) domain through to the C-terminal cytoplasmic tail (G1145), are important in mediating CR3 outside-in signals to the cytoplasm. As seen with R77H, variants can affect function even if not in classically identified-ligand binding or intracellular signaling domains. This effect may be due to necessary interactions between CD11b and CD18 or due to the requirement for large conformational changes occurring during integrin activation/signaling [46].

The ”hypothesis-free” GWAS method has proven successful in highlighting novel disease-associated polymorphisms for further functional analysis, but lacks statistical power to prove the association of most rare variants with disease. Recent approaches have identified rare variants in disease-associated loci very successfully, but estimating their contribution to disease risk has proven difficult. Conclusions are leading toward the notion that rare variants contribute little toward genetic variance [15]. We demonstrated that once the functional consequence of a common variant has been clearly elucidated, this knowledge can be reapplied to provide proof of a role for additional candidate rare variants at the same locus. In a relatively small resequencing project, we used this approach to identify two functional rare variants at the *ITGAM* locus. This study suggests that further screening of *ITGAM* for rare variants in a larger cohort of SLE cases promises to be worthwhile. If an association-based approach alone were applied in this study, there would be no evidence of any potential contribution of these rare variants to SLE pathogenesis. However, it is not possible to disregard these variants, given their genetic location, conservation, and deleterious effects on receptor function. If each of the 52 known SLE-susceptibility loci contains a similar high number of modest effect-size rare variants, then their contribution to the overall genetic component of disease susceptibility will be not inconsiderable. However, perhaps we currently lack the correct tools for estimating the contribution of rare variation to complex disease susceptibility.

## Conclusion

We have identified two novel rare variants in the SLE susceptibility gene, *ITGAM*, and demonstrated their functional effects. However, because of their extremely low frequencies, they are not statistically associated with disease risk. This study questions the current notion of dismissing the contribution of very rare variants on purely statistical analyses alone. Our results add further evidence to the functional importance of *ITGAM* in SLE pathogenesis through impaired phagocytosis.

## Abbreviations

CR3: Complement receptor 3; GWAS: genome-wide association study; LD: linkage disequilibrium; SLE: systemic lupus erythematosus.

## Competing interests

The authors declare that they have no competing interests.

## Authors’ contributions

ALR: study design, data collection and analysis, and manuscript drafting. ERAT: study design, data collection and analysis, and manuscript drafting. SB: data collection and analysis. LG: data collection and analysis. OO: data collection and analysis. TJA: study conception and critical revision of the manuscript. TJV: interpretation of data and critical revision of the manuscript. BR: study conception and design, interpretation of data, and critical revision of the manuscript. All authors read and approved the final manuscript.

## Supplementary Material

Additional file 1: Table S1 Primer sequences used to generate amplicons covering coding regions of *ITGAM* for 454 library preparation. Click here for file

Additional file 2: Figure S1 Rare variant validation by capillary sequencing. Electropherogram images showing the Sanger Sequencing validation of the two rare variants. Click here for file

Additional file 3: Table S2 Percentage phagocytosis of WT and variant CD11b-transfected COS-7 cells. Raw data comparing the percentage phagocytosis of WT and variant CD11b-transfected cells across five independent assays. Click here for file
