# Supplementary material for: Resequencing the susceptibility gene, ITGAM, identifies two functionally deleterious rare variants in systemic lupus erythematosus cases
Source: Arthritis Res Ther. 2014 May 21;16(3):R114. doi: 10.1186/ar4566 (PMC4060450; doi:10.1186/ar4566)
Supplement: Additional file 1: Table S1 — Primer sequences used to generate amplicons covering coding regions of ITGAM for 454 library preparation. [file ar4566-S1.pdf]

Table S1. Primer sequences used to generate amplicons covering coding regions of *ITGAM* for 454 library preparation.

Primer name	Sequence	Annealing temp (°C)	Amplicon size (bp)
ITGAM1F	GACGGGGTCTTATTATGTTGC	60	2761
ITGAM1R	ACATGGAACACAAACCTCACC		
ITGAMex1CF†	AAGACCAGGCAGGGCTATGT	60	294
ITGAMex1CR†	CCTACATATTCTGGGCCCTCT		
ITGAM2AF2	CCCAGCCATTGCTTTCTTTTA	60	732
ITGAM2AR2	CAAGTGGACGGAGTTGTTTTC		
ITGAM2BF	ATGGGGTCTGGCTATGTTACC	60	1930
ITGAM2BR	ACTTGCTGCAGCTTCAACTTC		
ITGAM3AF	GAGACGGGGTCCTATATTCTCA	60	1329
ITGAM3AR	ACTCCACTCCCACCTTTCACT		
ITGAM3BF	ACATGCCTGTAGTCCCAGGTA	60	599
ITGAM3BR	AAGGCCATTGAGACCAGAACT		
ITGAM3B2F†	CTAGTGTGTCATCTACTTGCATTTG	60	351
ITGAM3B2R†	GGCAACAGATAGCTGATACGG		
ITGAM4F	TGGATACAGGAAGAGGCAAGG	60	4089
ITGAM4R2	ATGCTGCTATAAAGGCACACG		
ITGAM5F	GGCAGCACATTCAAGTGATAA	58	798
ITGAM5R	AAACAATCCTCCCAACTCAGC		
ITGAM6F	CATTTCCCTTGCTCTTGAGG	58	1380
ITGAM6R	CCCCAAATAGGTTTTCTCTCC		
ITGAM7F	GCAGGTGGATTACTTTGAGACC	60	3957
ITGAM7R	GAGGCCACTCTTAAACACACC		
ITGAM8AiF	GTGTTGTTTGGGATTTGCAGT	60	711
ITGAM8AiR	TGCACGCACACACACATAGAT		
ITGAM8AiiF	GTGTCCAGCACACATTGGTTC	62	574
ITGAM8AiiR	CCAGTCCTAGCTCTCCCACTT		
ITGAM8AiiibF	AAGTGGGAGAGCTAGGACTGG	60	926
ITGAM8AiiibR	CTCACGATCAGGAGGTGGTTA		
ITGAM8BF	CAGTGTGACATCCCGTTCTTT	60	3606
ITGAM8BR	TGTTCACCTGTCAAACATCCA		

†Primers used for additional capillary sequencing.
