# Supplementary material for: Resequencing the susceptibility gene, ITGAM, identifies two functionally deleterious rare variants in systemic lupus erythematosus cases
Source: Arthritis Res Ther. 2014 May 21;16(3):R114. doi: 10.1186/ar4566 (PMC4060450; doi:10.1186/ar4566)

Figure S1. Rare variant validation by capillary sequencing

a) Reverse sequence of *ITGAM* exon 30 obtained from capillary sequencing. The solid arrow highlights the C/T heterozygous peak encoding the G1145S rare variant (codon change = GGT → AGT), and the dotted line highlights G/A heterozygous peak encoding the P1146S polymorphism (codon change = CCC → TCC).

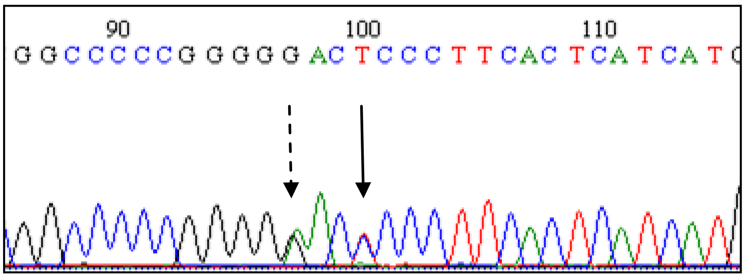

b) Forward sequence of *ITGAM* exon 21 obtained from capillary sequencing. The solid arrow highlights the T/G heterozygous peak encoding the F941V rare variant (codon change = TTC → GTC)

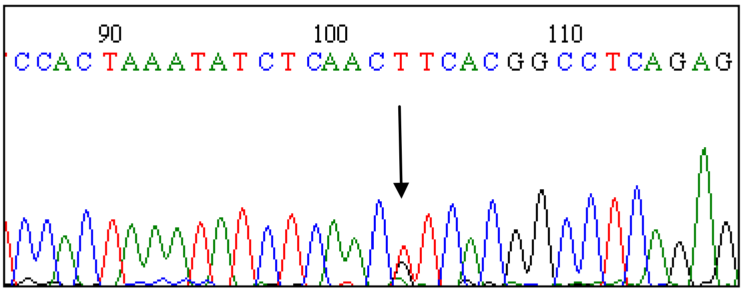

Supplement: Additional file 2: Figure S1 — Rare variant validation by capillary sequencing. Electropherogram images showing the Sanger Sequencing validation of the two rare variants. [file ar4566-S2.pdf]
