# Supplementary material for: Resequencing the susceptibility gene, ITGAM, identifies two functionally deleterious rare variants in systemic lupus erythematosus cases
Source: Arthritis Res Ther. 2014 May 21;16(3):R114. doi: 10.1186/ar4566 (PMC4060450; doi:10.1186/ar4566)
Supplement: Additional file 3: Table S2 — Percentage phagocytosis of WT and variant CD11b-transfected COS-7 cells. Raw data comparing the percentage phagocytosis of WT and variant CD11b-transfected cells across five independent assays. [file ar4566-S3.pdf]

Table S2. Percentage phagocytosis of WT and variant CD11b-transfected COS-7 cells

Raw data comparing the percentage phagocytosis of WT and variant CD11b-transfected cells across 5 independent assays

a)

	**Percentage Phagocytosis**				
***ITGAM* variant**	Assay 1	Assay 2	Assay 3	Assay 4	Assay 5
WT	10.62	11.62	13.53	12.73	12.17
M441T	14.32	8.98	7.22	16.04	10.19
A858V	19.50	8.13	10.75	18.10	12.84
G1145S	9.09	5.54	9.73	11.48	9.17

b)

	**Percentage Phagocytosis**				
***ITGAM* variant**	Assay 1	Assay 2	Assay 3	Assay 4	Assay 5
WT	8.4	20.7	14.3	12.11	15.57
F941V	5.91	10.0	2.20	2.92	5.59
